# The Use of the Kinetic Theory of Gases to Simulate the Physical Situations on the Surface of Autonomously Moving Parts During Multi-Energy Vibration Processing

**DOI:** 10.3390/ma12193054

**Published:** 2019-09-20

**Authors:** János Kundrák, Andrey V. Mitsyk, Vladimir A. Fedorovich, Michael Morgan, Angelos P. Markopoulos

**Affiliations:** 1 Institute of Manufacturing Science, University of Miskolc, 3515 Miskolc, Hungary; janos.kundrak@uni-miskolc.hu; 2 Kharkov Polytechnic Institute, Department of Integrated Engineering Techniques n.a. M.F. Semko, National Technical University, 61002 Kharkov, Ukraine; an.mitsyk@gmail.com (A.V.M.); fedvlad49@gmail.com (V.A.F.); 3 Engineering Research Institute, Liverpool John Moores University, Brownlow Hill, Liverpool L3 5UG, UK; m.n.morgan@ljmu.ac.uk; 4 Laboratory of Manufacturing Technology, School of Mechanical Engineering, National Technical University of Athens, 15772 Athens, Greece

**Keywords:** multi-energy vibration processing, dissipation of kinetic energy, pseudo-wave, movement of medium granules, movement of part, metal removal

## Abstract

The multi-energy vibration processing, namely the combination of different energies or forces acting on a free abrasive medium for grinding of metal parts, is becoming more used in finishing processes, in recent years. However, the complexity that is involved in the aforementioned process requires a careful look in the particularities of the process itself in general and the movement of the abrasive media, in particular. In this paper, the nature of the collective movement of abrasive granules between the independently oscillating surfaces of the reservoir and the processed parts is described. This study presents the dissipation of the kinetic energy of the granules in a pseudo-gas from the working medium granules. The motion of the medium granules near the part surface, which is caused by pseudo-waves initiated by vibrations of the working surfaces of the vibration machine reservoir, is demonstrated. Furthermore, the nature of the motion of the granules near the oscillating part surface is described. The analysis that is presented here permits the determination of metal removal quantity from the surface of the workpiece as a result of multi-agent group action of the vibrating reservoir surface and the processed part. The optimal conditions for the finishing process can be determined based on the analysis presented.

## 1. Introduction

In this paper, the results of the theoretical simulation of the situations that arise in the case of multi-energy vibration processing of parts are presented. Multi-energy vibratory finishing and grinding is a process that is organized by combining various processing schemes, energies, or forces acting on a free abrasive medium and autonomously moving parts placed in an oscillating vibration machine reservoir while using spindle devices and vibration manipulators. The present studies and reasoning are based on the assumption that the behavior of the granules of the working medium under the action of oscillations is analogous to the behavior of the gas. The physical situations of multi-energy vibration processing of autonomously moving parts significantly differ from situations when pseudo-gas from abrasive granules moves during the classical vibration processing, when the parts are placed “heaped up” or “fixed” in the reservoir of a vibration machine. This assumption is based on the conclusions of previous works [1,2,3], where it is asserted that, under the influence of autonomous oscillations of the working surfaces of the reservoir and the processed parts, the granules of the medium acquire significant kinetic energy and they begin to perform movements analogous to the motion of atoms or gas molecules. This is due to the fact that the working surfaces of the vibration machine reservoir, which form oscillatory movements in the pseudo-gas of abrasive granules, and the part move with different amplitudes and frequencies.

An analysis is given in [1] for the possibility of forming a compressed pseudo-gas layer from abrasive granules on the surface of the processed part that moves autonomously. This phenomenon is analogous to the effect of a shock wave in a conventional gas. It is shown how compaction of the pseudo-gas from abrasive granules leads to an intensification of the vibration processing. With the obtained relationships, it is possible to estimate the intensification value of the process, depending on the values of the speeds of the working surfaces of the vibrating machine and the surface of the processed part. However, solving the problem of improving the mechanisms and modes of vibration processing requires a more detailed analysis of the process, while taking into account the influence of the amplitude and frequency characteristics of the autonomous parts and oscillating walls of the vibration machine reservoir. Towards this goal, the works by Mediratta et al. [4] and Yang and Li [5] provide overviews of mass finishing processes, including multi-energy vibratory finishing and grinding. In the past, modeling with different methods was used to achieve an insight of advanced manufacturing processes [6,7]. The category of vibratory finishing is no exception and works while using Finite Elements and Discrete Elements methods can be found in the relevant literature [8,9]. The works by Hashimoto and Johnson [10], Sokolov and Krol [11,12], and Sokolov and Rasskazova [13] focus more on the equipment and tools of vibratory finishing. However, in the at hand paper, modelling is performed using the example of propagation of force action in the pseudo-gas from abrasive granules. Such a force action is accomplished from one oscillating infinite surface to another, which oscillates with a frequency and amplitude different from the first. At the same time, one surface plays the role of the wall of a vibration machine reservoir, and the other surface is the surface of the processed part. It is shown in [2,3] that the collective motion of a pseudo-gas that is caused by the motion of a flat or rounded surface is similar. Therefore, such a scheme allows for us to reveal the main theoretical regularities of the interaction of the abrasive granules activated by the oscillating walls of the reservoir with the surfaces of the autonomously moving processed parts.

## 2. Basic Provisions and Approaches

**Figure 1:**
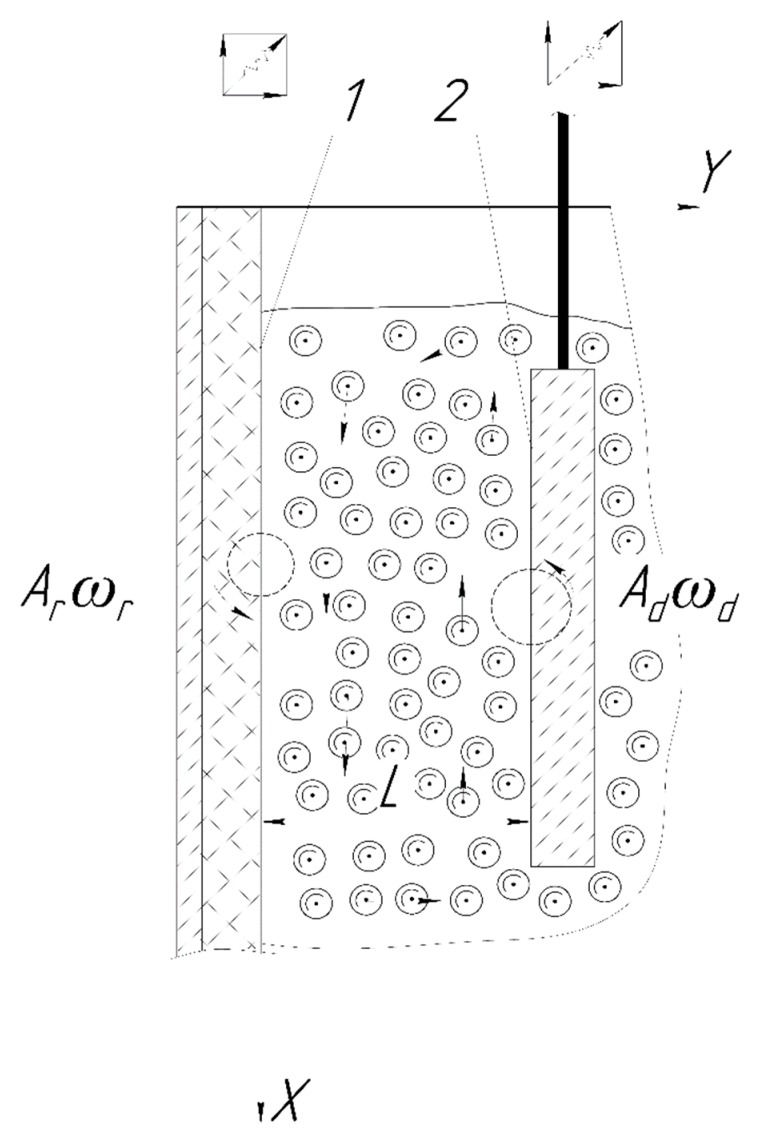
Oscillating surfaces filled with pseudo-gas from the abrasive granules.

The study shows two oscillating surfaces, between which there is a pseudo-gas from abrasive granules (Figure 1). The circles show the dynamics of any point of each of the surfaces. Differences in the radii of circles correspond to the difference in the amplitude of the motion of the surfaces. Directions of chaotically moving abrasive granules are indicated by the straight arrows. The frequencies of oscillations are also different. The coordinate system is chosen, so that the Y axis is horizontal and the X axis is vertical (Figure 1). This is due to the fact that the solutions for collective motion of an array of abrasive granules were obtained earlier [2] in this coordinate system. These expressions are rather cumbersome and their transformation when using a different coordinate system can lead to inaccuracies.

According to the scheme, the pseudo-gas fills an infinitely long strip of width (Figure 1). Each point of the left surface or for the wall of the vibration machine reservoir moves with a velocity, the components of which can be represented as:

(1) $$V_{r_{y}}=A_{r}\;\omega_{r}\cos\;\left(\omega_{r}t\right)$$

(2) $$V_{r_{x}}=A_{r}\;\omega_{r}\;\sin\;\left(\omega_{r}t\right)$$

Similarly, for the right surface or for the surface of the processed part, the components can be written:

(3) $$V_{d_{y}}=A_{d}\omega_{d}\;\cos\left(\omega_{d}t\right)$$

(4) $$V_{d_{x}}=A_{d}\;\omega_{d}\;\sin\left(\omega_{d}t\right)$$

Here, $V_{r_{x}},\;V_{r_{y}},\;V_{d_{x}},\;V_{d_{y}}$ are the components of the speeds of movement for any point of the reservoir surface and the processed part, directed along the axes and X, Y, respectively, $\omega_{r}$, $\omega_{d}$—circular frequency of oscillations of the walls reservoir and the part.

To solve the set task, we use the continuity Equation (5) and Navier-Stokes Equation (6) [14]:

(5) $$\frac{\partial \rho }{\partial t}+\mathrm{div}\left(\rho \bar{V}\right)=0$$

(6) $$\frac{d\bar{V}}{dt}=\bar{F}-\frac{1}{\rho }grad\;p+\nu \Delta \bar{V}+\left(\frac{\zeta }{\rho }+\frac{\nu }{3}\right)grad\;div\bar{V}$$

Here $\bar{V}$—speed of movement of an elementary volume of gas (liquid), $\bar{F}$—field strength of mass forces, ρ—gas density, ν=η/ρ and η—constant coefficients of kinematic and dynamic viscosity, and ζ—constant coefficient of the second or bulk viscosity.

After simplifications that are based on reasonable assumptions [2], the Navier-Stokes equations for finding the velocity components $V_{x}$ and $V_{y}$ have the form:

(7) $$\left\{ \begin{array}{l} \frac{\partial V_{x}}{\partial t}=F_{x}+\nu \frac{\partial^{2}V_{x}}{\partial y^{2}}\\ \frac{\partial V_{y}}{\partial t}=F_{y}-\frac{1}{\rho }\frac{\partial p}{\partial y}+\nu \frac{\partial^{2}V_{y}}{\partial y^{2}} \end{array}\right.$$

Neglecting the mass forces, that is, the gravitational forces, we obtain two linear independent equations with respect to the two components of the velocities of the motion of the pseudo-gas from the abrasive granules.

Equation (7) describe the propagation of vibrations of the abrasive granules in the pseudo-gas. The oscillations are transmitted from the working surfaces of the reservoir and the surfaces of the processed parts. This is described by the Equations (1)–(4). In acoustics, the propagation of oscillations, in any medium, is called a wave. This definition is confirmed by the solution of system (7), as shown in [2]. In the case when only the working reservoir surface exerts a force action on the mass of the abrasive, the solution for the components of collective motion is as follows:

(8) $$\left\{ \begin{array}{ll} V_{x}\left(y,\;t\right)= & -A\omega e^{-\sqrt{\omega /2\nu }y}\;\cos\left(\omega t-\sqrt{\frac{\omega }{2\nu }}y\right)\\ V_{y}\left(y,\;t\right)= & A\omega [e^{-\sqrt{\omega /2\nu }y}((1+\frac{2}{3}kA\sqrt{\frac{\omega }{2\nu }})\;\cos(\omega t-\sqrt{\frac{\omega }{2\nu }}y)+\\ & +\frac{2}{3}kA\sqrt{\frac{\omega }{2\nu }}\;\sin(\omega t-\sqrt{\frac{\omega }{2\nu }}y))-\frac{2}{3}kA\sqrt{\frac{\omega }{2\nu }}\;e^{-2\sqrt{\omega /2\nu }y}\times \\ & \times (\cos(\omega t-\sqrt{\frac{\omega }{2\nu }}y)+\sin(\omega t-2\sqrt{\frac{\omega }{2\nu }}y))] \end{array}\right.$$

These expressions are derived from the hypothesis that the velocity of the pseudo-gas layer in close proximity to the reservoir working surface is equal to the velocity of the reservoir [15], and the pressure on a vibrating surface is determined from the relation $p\sim \rho kT\sim \rho V^{2}$.

It can be seen from Equation (8) that the components of the rate of collective motion of the pseudo-gas from the abrasive granules are described by a superposition of functions characterizing the damped wave motion occurring at velocities $\sqrt{2\nu \omega }$ and $0.5\;\sqrt{2\nu \omega }$ that depend on the oscillation frequency of the reservoir. Due to this, as well as taking into account the linearity of the Equation (7), the motion of the abrasive granules can be regarded as a superposition of damped waves. Consequently, the movement of the abrasive working medium under the influence of oscillations of the vibration machine between two independent solid surfaces of the reservoir and the part can be considered as the interaction of acoustic waves that are generated by their motion. This approach allows for us to determine the interaction of the working medium with the surface of the processed part, while using acoustic approximation.

### 2.1. Boundary Conditions

Let us consider the surface of the processed part (Figure 1). In the reflection of a plane acoustic wave moving perpendicular to the elastic surface of the processed part, the product of the densities and velocities of sound in the medium of propagation of the wave and in the material of the part play an important role. In our case, this is $\rho_{cg}$, $c_{cg}$ and $\rho_{d}$, $c_{d}$. Here, $\rho_{cg}$, $c_{cg}$ corresponds to the average density of the collective of granules and the speed of propagation of disturbances in it, that is, the speed of sound in the pseudo-gas from the abrasive particles. The notation $\rho_{d}$, $c_{d}$ corresponds to the density of the material of the processed parts and the speed of sound in it. The density of metal processed parts exceeds the density of the material of the granules and, thus, exceeds the average density of the pseudo-gas from the abrasive granules. It is assumed that the speed of sound in the metal of the part is several kilometers per second, but the speed of sound in the pseudo- gas cannot exceed the speed of movement of the abrasive granules. At a maximum oscillating frequency of vibration machine of about 70 Hz and the greatest oscillation amplitude of 5 mm, it is slightly more than 2 m/s. Thus, we can write that $\rho_{cg}c_{cg}\ll \rho_{d}c_{d}$. In this case, the wave that is reflected from the surface of the processed part changes the phase of oscillations by half the period of oscillations of the incident wave. The amplitude of oscillations of the reflected wave, without taking into account inelastic losses, is equal to the amplitude of the oscillations of the incident wave [16].

Thus, on the surface of the processed part, the boundary conditions are determined by a superposition of the incident and reflected waves, besides the thermodynamic parameters of the pseudo-gas. Here, as in [1], we will take the loss of kinetic energy upon impact of the abrasive granules with the surface of the processed part with the help of the recovery coefficient into account [15].

### 2.2. Surface of the Processed Part

Let us consider the process of collision of a moving single granule with the moving surface of the processed part in more detail.

**Figure 2:**
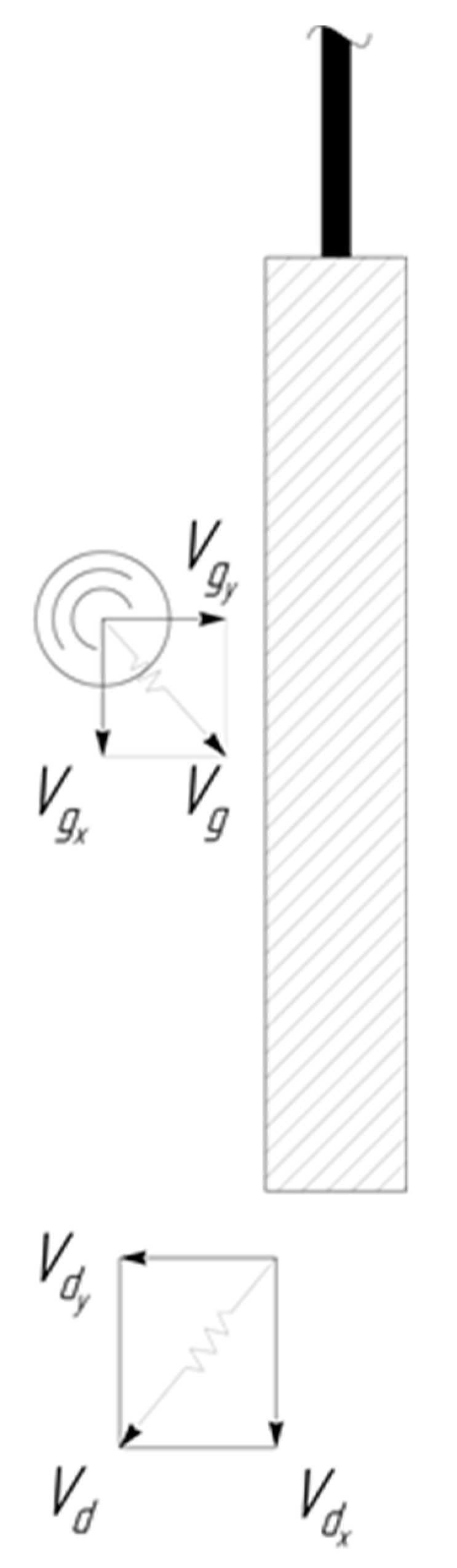
Collision of the moving granule with the surface of the processed part.

Let us schematically show the arrival of abrasive granule to the surface of the processed part (Figure 2). The speed of approach of the granule and the part is the geometric sum of the velocities of both. Consequently, the component of the approach velocity, the granule and the part directed along axis Y, will be $V_{g_{y}\Sigma }=-V_{d_{y}}+V_{g_{y}}$, and the component directed along the axis is X–$V_{g_{x}\Sigma }=-V_{d_{x}}+V_{g_{x}}$. According to [17], the component of the rebound velocity directed along the Y axis can be written in the coordinate system moving together with the processed part, in the form $V_{g_{y}\mathrm{reb}}=V_{g_{y}\Sigma }\;\beta$, where $\beta =\sqrt{\frac{1-f}{1+f}}$ is the recovery coefficient, and f is the coefficient of dry friction. Thus, for the component of the rebound velocity directed along the Y axis in the coordinate system that is associated with the surface of the processed part, we can write:

(9) $$V_{g_{y}\;\mathrm{reb}}=\left(V_{d_{y}}-V_{g_{y}}\right)\;\beta$$

**Figure 3:**
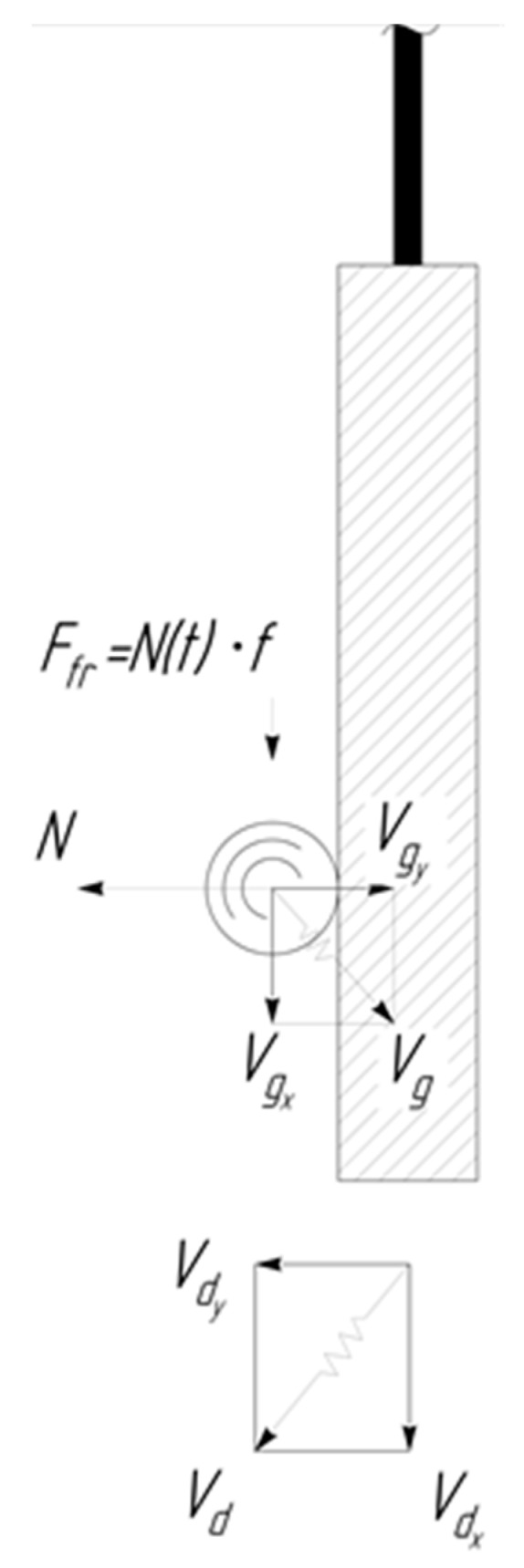
Change in the velocity component of the granule along the X axis after its collision with the processed part $F_{fr}=N\left(t\right)f$.

The component of the velocity of the granule after the rebound along the X axis can be determined from the following considerations (Figure 3).

The collision process, by virtue of the fact that the mass of the part exceeds by several orders the magnitude the granule mass—$m_{g}$, can be described in a coordinate system moving with the part at the moment of collision by the following system of equations:

(10) $$\left\{ \begin{array}{l} m_{g}\;\frac{dV_{g_{y}\;\Sigma }}{dt}=N\left(t\right)\\ m_{g}\;\frac{dV_{{}_{g_{x}\;\Sigma }}}{dt}=f\;N\left(t\right) \end{array}\right.$$

Here, N(t) is the reaction force of the plane when it hits the processed part.

The solution of the system of Equation (10) is difficult because of the unknown function N(t). However, finite expressions for the components of the motion velocities can be obtained while using the law of conservation of momentum [14]. We equate the change in the vertical component of the number of granule movement as a result of the collision, to the impulse of reaction force of the part, which acts into the granule during the impact—Δt:

(11) $$\int_{0}^{\Delta t}N\left(t\right)\;d\tau =m_{g}\left(V_{d_{y}}-V_{g_{y}}\right)\;\left(\beta +1\right)$$

On the other hand, the reaction force of the part during the impact will cause a change in the amount of movement of the granule in the direction along the X axis due to the frictional force:

(12) $$f\;\int_{0}^{\Delta t}N\left(t\right)\;d\tau =m_{g}\;\Delta V_{g_{x}}$$

In the case where the component of the speed of the granule along the X axis is opposite to the direction of the component of the wall, the speed of the granule will decrease, whereas it will increase in the opposite case. The maximum possible change in the horizontal component of the granule can be expressed from the Equations (11) and (12) by the formula:

(13) $$\Delta V_{g_{x}\;\max}=f\left(V_{d_{y}}-V_{g_{y}}\right)\left(\beta +1\right)\;\mathrm{sign}\left(V_{d_{x}}-V_{g_{x}}\right)$$

### 2.3. Working Surface of the Vibrating Machine Reservoir

The working reservoir surface is lined with hard rubber (Figure 1). The Young’s modulus of shock rubber absorbers is ≈10 MPa [18]. It is known that if the natural oscillation frequency of the “rubber lining-granule” system is much higher than the oscillation frequency of the vibrating machine, then the velocity of the abrasive granule after the collision with the working reservoir surface should not depend on its speed before collision [19]. The cyclic frequency of the “rubber lining-granule” system is equal to the oscillation frequency of the spring pendulum and it is determined by the relation $\omega_{{}_{0}}\approx \sqrt{k/m}$ [14]. Here, k is the hardness coefficient of the rubber. In our case, we have $k\approx 10^{4}$ N/m. The mass of the granule is about one gram. Then the oscillation frequency will be $\omega_{{}_{0}}\geq 3\cdot 10^{3}$ rad/s. This value is almost an order of magnitude greater than the maximum circular vibrational frequency of the vibrating machine. This frequency will be about 440 rad/s. Thus, when calculating the propagation of force action from the working reservoir surface by the pseudo-gas from the abrasive, the granules’ velocities prior to impact against it can be ignored.

Real boundary conditions consist in the fact that the oscillatory movements of the working medium, together with the surface of the processed part and the reservoir, do not occur during the whole oscillation period. At some moment of time $t_{{}_{0}}=\alpha_{{}_{0}}/\omega$, when one of the surfaces is removed from the granules, tearing off of the granule mass takes place, that is, the components of the velocity of the abrasive granules along the Y axis become smaller than the components of the surface velocity of the reservoir along this axis. In this case, the movement of the mass of the abrasive, without regard for gravity, will be rectilinear. Such rectilinear motion is retained until time $t^{*}=\alpha^{*}/\omega$, when the reservoir surface during its movement again comes into contact with the granules of the working medium. The moments of separation and pick-up will be different for the working surfaces of the reservoir and the processed part, for the left (reservoir wall) and for the right (part wall) planes.

## 3. Collective Movement of the Abrasive Granules Between the Independently Oscillating Surfaces of the Reservoir and the Processed Part

Earlier, it was shown that the collective motion of the mass of the working medium under the influence of vibrations of the vibrating machine between two autonomously moving solid surfaces can be considered as the propagation of damped acoustic waves generated by their motion. In their movement between the two surfaces these waves receive energy, mainly from the working surface of the vibrating machine reservoir and partly from the oscillating surface of the processed part. The dissipation of energy of waves occurs due to inelastic collisions of the granules with each other during the propagation of a force pulse into the working medium. The efficiency of this energy is minimal. The second (useful) channel for absorbing the energy of surface vibrations is the removal of metal from the surface of the processed part.

Thus, it is necessary to determine initially the characteristics of the waves initiated by both surfaces, taking both energy absorption channels into account to clarify the pattern of motion of the medium granules between two surfaces.

### 3.1. Movement of the Collective of the Abrasive Granules Under the Action of the Working Surfaces of the Reservoir

From the foregoing, it follows that the motion of the granules can be regarded as a superposition of damped waves generated by independent vibrational motion of the working surfaces of the reservoir and the surface of the processed part. Near the surface of the vibrating machine reservoir, the solution of Equation (7) is described by the relationships that are presented in the work [2] with an allowance for real boundary conditions. The movement of the abrasive granules caused by the oscillations of the working surfaces of the reservoir, according to the work [2], is described by the following relationships:

(14) $$\left\{ \begin{array}{l} U_{s\sum }=\frac{a_{0}}{2}+\sum_{k=1}^{\infty }\left(W_{s_{x}k}\left(y,t\right)+V_{s_{x}k}\left(y,t\right)\right)\\ V_{s\sum }=\sum_{k=1}^{\infty }\left(V_{s_{y}k}\left(y,t\right)+W_{s_{y}k}\left(y,t\right)\right) \end{array}\right.$$

Here, $U_{\Sigma }$ and $V_{\Sigma }$ are the components of the motion of the granules of the working medium along the X and Y axes, respectively.

The terms of the series in Equation (14) are described by the following dependences:

(15) $$\left\{ \begin{array}{ll} V_{s_{x}k}\left(y,t\right)= & -b_{k}^{U}l^{-\sqrt{2\omega k/\nu_{k}}y}\sin\left(\omega kt-\sqrt{\frac{2\omega k}{\nu_{k}}}y\right)\\ V_{s_{y}k}\left(y,t\right)= & -a_{k}^{V}[l^{-\sqrt{\omega k/\nu_{k}}y}(\left(1+\frac{2}{3}l\frac{a_{k}^{V}}{\omega k}\sqrt{\frac{2\omega k}{\nu_{k}}}\right)\;\cos\left(\omega kt-\sqrt{\frac{2\omega k}{\nu_{k}}}y\right)+\\ & +\frac{2}{3}l\frac{a_{k}^{V}}{\omega k}\sqrt{\frac{2\omega k}{\nu_{k}}}\;\sin\left(\omega kt-\sqrt{\frac{2\omega k}{\nu_{k}}}y\right))-\frac{2}{3}l\frac{a_{k}^{V}}{\omega k}\;\sqrt{\frac{2\omega k}{\nu_{k}}}l^{-2\sqrt{2\omega k/\nu_{k}}y}\times \\ & \times \left(\cos\left(\omega kt-2\sqrt{\frac{2\omega k}{\nu_{k}}}y\right)+\sin\left(\omega kt-2\sqrt{\frac{2\omega k}{\nu_{k}}}y\right)\right)] \end{array}\right.$$

(16) $$\left\{ \begin{array}{ll} W_{s_{x}k}\left(y,t\right)= & -a_{k}^{U}l^{-\sqrt{2\omega k/\nu_{k}}y}\cos\left(\omega kt-\sqrt{\frac{2\omega k}{\nu_{k}}}y\right)\\ W_{s_{y}k}\left(y,t\right)= & b_{k}^{V}[l^{-\sqrt{\omega k/\nu_{k}}y}(\left(1+\frac{2}{3}l\frac{b_{k}^{V}}{\omega k}\sqrt{\frac{2\omega k}{\nu_{k}}}\right)\;\;\sin\left(\omega kt-\sqrt{\frac{2\omega k}{\nu_{k}}}y\right)-\\ & -\frac{2}{3}l\frac{b_{k}^{V}}{\omega k}\sqrt{\frac{2\omega k}{\nu_{k}}}\;\cos\left(\omega kt-\sqrt{\frac{2\omega k}{\nu_{k}}}y\right))+\frac{2}{3}l\frac{b_{k}^{V}}{\omega k}\sqrt{\frac{2\omega k}{\nu_{k}}}l^{-2\sqrt{2\omega k/\nu_{k}}y}\times \\ & \times \left(\cos\left(\omega kt-2\sqrt{\frac{2\omega k}{\nu_{k}}}y\right)-\sin\left(\omega kt-2\sqrt{\frac{2\omega k}{\nu_{k}}}y\right)\right)] \end{array}\right.$$

The coefficients $a_{k}^{V},\;a_{k}^{U}$ and $b_{k}^{V},\;b_{k}^{U}$ are expressed, as follows:

(17) $$a_{s0}^{U}=-\frac{2A_{s}}{T}\left[\left(\alpha_{s}{}^{*}-\alpha_{s0}\right)\sin\alpha_{s0}+\cos\alpha_{s}{}^{*}-\cos\alpha_{s0}\right]$$

(18) $$a_{s0}^{V}=0$$

(19) $$a_{s1}^{U}=-\frac{2A_{s}}{T}\left[\frac{1}{4}\left(\cos2\alpha_{s}{}^{*}-\cos2\alpha_{s0}\right)+\sin\alpha_{s0}\left(\sin\alpha_{s}{}^{*}-\sin\alpha_{s0}\right)\right]$$

(20) $$b_{s1}^{V}=-\frac{2A_{s}}{T}\left[\frac{1}{4}\left(\cos2\alpha_{s}{}^{*}-\cos2\alpha_{s0}\right)+\cos\alpha_{s0}\left(\cos\alpha_{s0}-\cos\alpha_{s}{}^{*}\right)\right]$$

(21) $$a_{s0}^{U}=-\frac{2A_{s}}{T}\left[\left(\alpha_{s}{}^{*}-\alpha_{s0}\right)\sin\alpha_{s0}+\cos\alpha_{s}{}^{*}-\cos\alpha_{s0}\right]$$

(22) $$a_{s0}^{V}=-\frac{2A_{s}}{T}\left[\left(\sin\alpha_{s0}-\sin\alpha_{s}{}^{*}\right)+\left(\alpha_{s}{}^{*}-\alpha_{s0}\right)\cos\alpha_{s0}\right]$$

(23) $$\begin{array}{ll} a_{sk}^{U}= & -\frac{2A_{s}}{T}[\frac{1}{2\left(k+1\right)}\;(\cos\left(\alpha_{s}{}^{*}\left(k+1\right)\right)-\\ & -\cos\left(\alpha_{s0}\left(k+1\right)\right))+\frac{1}{2\left(1-k\right)}(\cos\left(\alpha_{s}{}^{*}\left(1-k\right)\right)-\\ & -\cos\left(\alpha_{s0}\left(1-k\right)\right))+\frac{\sin\alpha_{s0}}{k}\left(\sin\left(k\alpha_{s}{}^{*}\right)-\sin\left(k\alpha_{s0}\right)\right)]\; \end{array}$$

(24) $$\begin{array}{ll} b_{sk}^{U}= & -\frac{2A_{s}}{T}[\frac{1}{2\left(k+1\right)}(\sin\left(\alpha_{s}{}^{*}\left(k+1\right)\right)-\\ & -\sin\left(\alpha_{s0}\left(k+1\right)\right))+\frac{1}{2\left(1-k\right)}(\sin\left(\alpha_{s0}\left(1-k\right)\right)-\\ & -\sin\left(\alpha_{s}{}^{*}\left(1-k\right)\right))-\frac{\sin\alpha_{s0}}{k}\left(\cos k\alpha_{s}{}^{*}-\cos k\alpha_{s0}\right)] \end{array}$$

(25) $$\begin{array}{ll} a_{sk}^{V}= & \frac{2A_{s}}{T}[\frac{1}{2\left(k+1\right)}\;(\sin\left(\alpha_{s0}\left(k+1\right)\right)-\\ & -\sin\left(\alpha_{s}{}^{*}\left(k+1\right)\right))+\frac{1}{2\left(1-k\right)}(\sin\left(\alpha_{s0}\left(1-k\right)\right)-\\ & -\sin\left(\alpha_{s}{}^{*}\left(1-k\right)\right))+\frac{\cos\alpha_{s0}}{k}\left(\sin\left(k\alpha_{s}{}^{*}\right)-\sin\left(k\alpha_{s0}\right)\right)]\; \end{array}$$

(26) $$\begin{array}{ll} b_{sk}^{V}= & \frac{2A_{s}}{T}[\frac{1}{2\left(k+1\right)}\;(\cos\left(\alpha_{s}{}^{*}\left(k+1\right)\right)-\\ & -\cos\left(\alpha_{s0}\left(k+1\right)\right))+\frac{1}{2\left(1-k\right)}(\cos\left(\alpha_{s0}\left(1-k\right)\right)-\\ & \cos\left(\alpha_{s}{}^{*}\left(1-k\right)\right))+\frac{\cos\alpha_{s0}}{k}\left(\cos\alpha_{s0}-\cos\alpha_{s}{}^{*}\right)] \end{array}$$

The Equations (23)–(26) that were obtained as a result of the transformations are valid for the values of k=2, 3, 4 … ∞. For values of k=1 they have an uncertainty, therefore these relations are written separately—Equations (19) and (20).

The coefficients $a_{1}^{U}$ and $b_{1}^{V}$, having a singularity in previous Equations (17)–(26), can be obtained while using L’Hôpital’s rule [20]. Afterwards, we get:

(27) $$a_{s1}^{U}=-\frac{2A_{s}}{T}\left[\frac{1}{4}\left(\cos2\alpha_{s}{}^{*}-\cos2\alpha_{s0}\right)+\sin\alpha_{s0}\left(\sin\alpha_{s}{}^{*}-\sin\alpha_{s0}\right)\right]$$

(28) $$b_{s1}^{V}=-\frac{2A_{s}}{T}\left[\frac{1}{4}\left(\cos2\alpha_{s}{}^{*}-\cos2\alpha_{s0}\right)+\cos\alpha_{s0}\left(\cos\alpha_{s0}-\cos\alpha_{s}{}^{*}\right)\right]$$

For the coefficients $a_{1}^{V}$ and $b_{1}^{U}$, the application of L’Hôpital’s rule gives an incorrect result. Therefore, it is necessary to integrate Equations (27) and (28) for the value of k=1. As a result, we get the following:

(29) $$\begin{array}{ll} b_{s1}^{U}= & -\frac{2A_{s}}{T}[\frac{1}{2}\left(\alpha_{s0}-\alpha_{s}{}^{*}\right)+\pi +\frac{1}{4}\left(\sin2\alpha_{s}{}^{*}-\sin2\alpha_{s0}\right)+\\ & +\sin\alpha_{s0}\left(\cos\alpha_{s}{}^{*}-\cos\alpha_{s0}\right)] \end{array}$$

(30) $$\begin{array}{ll} a_{p1}^{V}= & \frac{2A_{s}}{T}[\frac{1}{2}\left(\alpha_{p0}-\alpha_{p}{}^{*}\right)+\pi +\frac{1}{4}\left(\sin2\alpha_{p0}-\sin2\alpha_{p}{}^{*}\right)+\\ & +\cos\alpha_{p0}\left(\sin\alpha_{p}{}^{*}-\sin\alpha_{p0}\right)] \end{array}$$

Thus, the final expressions for the real components of the horizontal and vertical velocity components U(t) and V(t) on the working reservoir surface have the form:

(31) $$U_{s\Sigma }\left(t\right)=\frac{a_{s0}^{U}}{2}+\sum_{k=1}^{\infty }\left(a_{sk}^{U}\;\cos\left(k\omega t\right)+b_{sk}^{U}\;\sin\left(k\omega t\right)\right)$$

(32) $$V_{s\Sigma }\left(t\right)=\sum_{k=1}^{\infty }\left(a_{sk}^{V}\;\cos\left(k\omega t\right)+b_{sk}^{V}\;\sin\left(k\omega t\right)\right)$$

### 3.2. Movement of the Abrasive Granules of the Working Medium Near the Surface of the Processed Part

The movement of the surface of a part is described by the following relationships:

(33) $$V_{dy}=A_{d}\omega_{d}\;\cos\left(\omega_{d}t+\varphi \right)$$

(34) $$V_{dx}=A_{d}\;\omega_{d}\;\sin\left(\omega_{d}t+\varphi \right)$$

Here, φ is the phase difference between the oscillations of the reservoir working surface and the processed part.

According to the adopted model of acoustic approximation (see Section 2), the pressure near the working reservoir surface will be composed of three components [16]. The movement of the surface of the vibrating machine reservoir creates the pressure of the pseudo-gas and the surfaces of the reservoir and the processed part create the acoustic pressures. Thus, we can write the following relation:

(35) $$\rho_{cg}\;\frac{{\langle V_{x}\rangle }^{2}}{3}=\rho_{pg}\;\frac{{\langle V_{sd}\rangle }^{2}}{3}+\rho_{cg}\langle V_{x}\rangle \;\sqrt{\frac{\gamma }{3}}\;\langle V_{sda}\rangle +\rho_{cg}\;\langle V_{x}\rangle \;\sqrt{\frac{\gamma }{3}}\;\langle V_{da}\rangle$$

where $\langle V_{sda}\rangle$ is the root-mean-square velocity of chaotic movement of granules in a pseudo-gas created near the surface of a part by the oscillating surface of a vibrating machine reservoir; $\langle V_{x}\rangle$ is the root-mean-square velocity of the granules in the pseudo-gas, which was acquired as a result of the total action of acoustic waves and by the chaotic movement of the pseudo-gas granules; $\langle V_{sda}\rangle =\sqrt{\frac{1}{t}\int_{0}^{t}V_{sda}^{2}\left(t\right)\;dt}$—is the time-averaged velocity of oscillatory motion of the granules under the action of an acoustic wave produced by the reservoir surface near the part surface; $\langle V_{da}\rangle =\sqrt{\frac{1}{t}\int_{0}^{t}V_{da}^{2}\left(t\right)\;dt}$ is the time-averaged velocity of the granules near the part surface acting as an oscillating plane acoustic radiator; $\rho_{cg},\;\gamma$ is the density of the pseudo-gas and the adiabatic index, respectively, equal to 5/3 in our case.

Solving the Equation (35) with respect to the unknown root-mean-square velocity $\langle V_{x}\rangle$, we obtain the relation:

(36) $$\langle V_{x}\rangle =\sqrt{{\langle V_{sd}\rangle }^{2}+\frac{3}{4}\;\gamma \;{\left(\langle V_{sda}\rangle +\langle V_{da}\rangle \right)}^{2}}+\frac{\sqrt{3\gamma }}{2}\;\left(\langle V_{sda}\rangle +\langle V_{da}\rangle \right)$$

#### 3.2.1. Dissipation of the Kinetic Energy of the Granules in the Pseudo-Gas from the Granules of the Working Medium

To determine the decrease in the rates of chaotic and wave motion of the granules that are associated with friction between granules during their collisions, we use the results of [21]. It is shown there that the mean free run length of granules in a pseudo-gas from the abrasive granules is expressed by the following relationship:

(37) $$l=\frac{\pi R\left(1+C{\langle V\rangle }^{2}\right)}{3r\sqrt{2\pi }}$$

where R is the radius of the granule, 〈V〉 is the root-mean-square velocity of the granule, r is the laying ratio of the granules (r=0.66).

The constant C is proportional to the value of the ratio of the density of the material of the granules to the pressure that was created by them during the circulation movement. It does not depend on the density of the granule material. Its value in the International System of Units SI can take values in the range 10–30.

The loss of kinetic energy of the granule in inelastic collisions with the rest of the abrasive granules is determined by the relation:

(38) $$\langle \Delta E_{n}\rangle =\langle E_{0}\rangle {\left(1-\varepsilon \right)}^{n}$$

where $E_{n}$ is the kinetic energy after the n-number collision of granules in their translational motion; $E_{0}$ the initial kinetic energy of granules; and, n the number of granules collisions during its chaotic motion in the pseudo-gas.

The value ε is equal to the loss of kinetic energy for one inelastic collision between the granules and, according to [21], taking into account the recovery coefficient [17] (see Section 2.2), it is determined by the following relation:

(39) $$\begin{array}{ll} \varepsilon = & 7\left(\frac{\cos^{3}\alpha }{3}-\cos\alpha \right){|}_{0}^{{\mathrm{tn}}^{-1}\left(7\beta f\right)}+\\ & +f\left(\frac{7}{2}\;\times \frac{\cos^{3}\alpha }{3}{|}_{\frac{\pi }{2}}^{{\mathrm{tn}}^{-1}\left(7\beta f\right)}-\frac{\sin^{3}\alpha }{3}{|}_{{\mathrm{tn}}^{-1}\left(7\beta f\right)}^{\frac{\pi }{2}}\right) \end{array}$$

Using the Equations (38) and (39), we can express the velocity of chaotic movement of the granules as a function of the velocity of the working reservoir surface and the distance from it:

(40) $$\frac{{\langle V\rangle }^{2}}{{\langle V_{0}\rangle }^{2}}={\left(1-\varepsilon \right)}^{n}$$

The number of collisions –n when abrasive granules transfer the pulse during their chaotic motion for some distance – r occurs randomly. Therefore, the relationship between n, r and the distance at which momentum is transmitted –λ must be determined by the relation $r^{2}=\lambda^{2}n$ [22]. The value λ is equal to the sum of the mean free run length of the granule in the pseudo-gas and the diameter of the granule. It is λ=d+l Thus, Equation (40) can be written in the following form:

(41) $${\langle V_{sd}\rangle }^{2}/{\langle V_{0}\rangle }^{2}={\left(1-\varepsilon \right)}^{{}^{y^{2}/{\left(d+\frac{\left(1+\;C{\langle V_{HB}\rangle }^{2}\right)}{6\sqrt{2}\;\cdot 0.66}d\right)}^{2}}}$$

Here, the variable y means the distance of the point where the root-mean-square velocity of the chaotic movement of the granules is determined in the pseudo-gas from the surface of the vibrating machine reservoir, $\langle V_{0}\rangle$ is the root-mean-square velocity of chaotic motion near the working surface of the reservoir.

**Figure 4:**
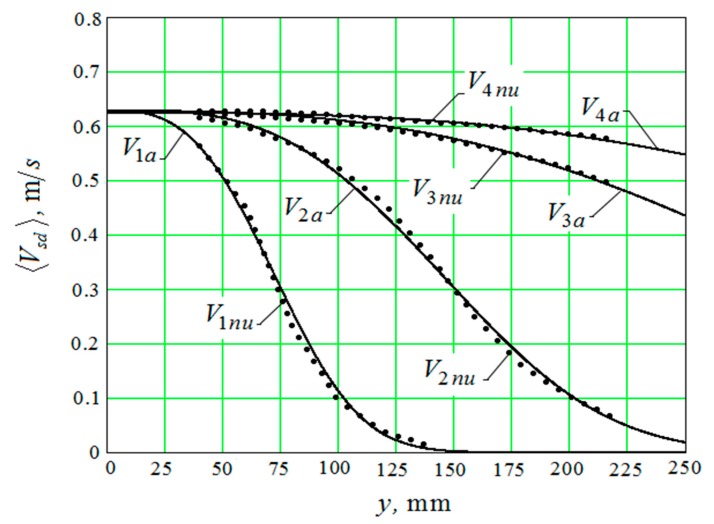
The results of the numerical solution of Equation (41).

Ratio (41) also contains the variable d– the diameter of the abrasive granule. Equation (41) can only be solved with respect to $\langle V_{sda}\rangle$ numerically. The result of the numerical solution of Equation (41) for different values of the diameter of the granules (Figure 4) on the curves is indicated by dots. The graphs that were obtained with an approximate formula are represented by continuous lines.

The curves $V_{{}_{1}}{}_{nu},\;V_{2}{}_{nu},\;V_{3}{}_{nu},\;V_{4}{}_{nu}$ correspond to diameters of abrasive granules of 10 mm; 20 mm; 40 mm; and, 60 mm and they are an approximation to the numerical data that were obtained by the Equation (42).

The approximate formula, which is necessary for further calculations, has the form: $V_{1\;a}$

(42) $$\frac{\langle V_{sd}\rangle }{A_{s}\;\omega_{s}}≃e^{-f_{1}\left(d\right)\cdot {\left(y-f_{2}\left(d\right)\right)}^{2.99}}$$

$f_{1}\left(d\right)$ and $f_{2}\left(d\right)$ are the functions of the diameter of the abrasive granules, which are the result of interpolation of the numerical solution of Equation (41). Both functions have the following form:

(43) $$\begin{array}{ll} f_{1}\left(d\right)= & 1800\cdot [e^{-400\;\left(d-0.008\right)}+0.001789\;e^{8.5\;\left(d-0.035\right)}\\ & +0.04\;e^{-136\;\left(d-0.05\right)}] \end{array}$$

(44) $$f_{2}\left(d\right)=0.0103-15.9\;{\left(d-0.035\right)}^{2}$$

**Figure 5:**
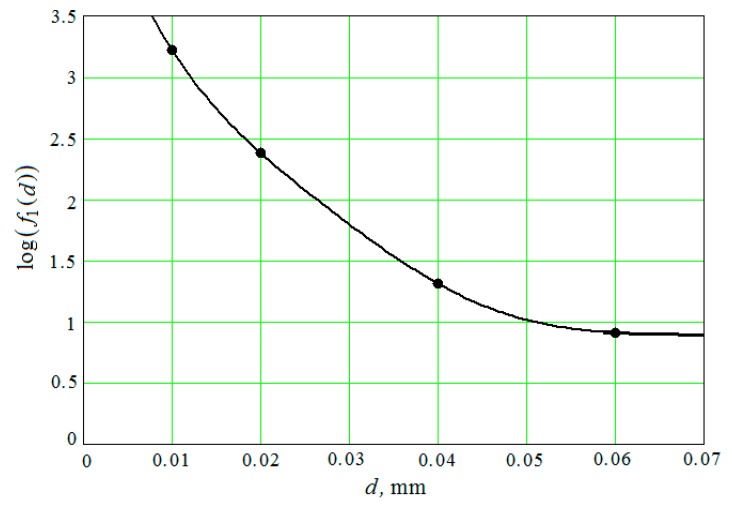
The decimal logarithm $f_{1}(d)$ (continuous curve) and the results of the numerical solution of Equation (41) (points).

**Figure 6:**
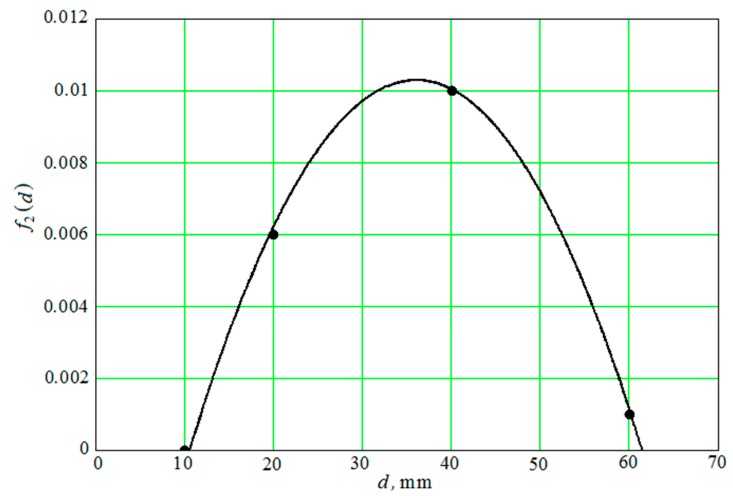
The function $f_{2}(d)$ (continuous curve) and the results of the numerical solution of Equation (41) (points).

The degree of coincidence of functions $f_{1}\left(d\right)$ and $f_{2}\left(d\right)$ and the calculated data (Figure 5 and Figure 6) was obtained by numerically solving Equation (41) for four values of the diameters of the abrasive granules of 10 mm; 20 mm; 40 mm; and, 60 mm.

The root-mean-square relative deviation of the values that were calculated by the Equation (42) from the values obtained by the numerical solution of the Equation (41) is less than 6%.

#### 3.2.2. The Motion of the Medium Granules Near the Part Surface Caused by Pseudo-Waves Initiated by Oscillations of the Working Surfaces of the Vibrating Machine Reservoir

**Figure 7:**
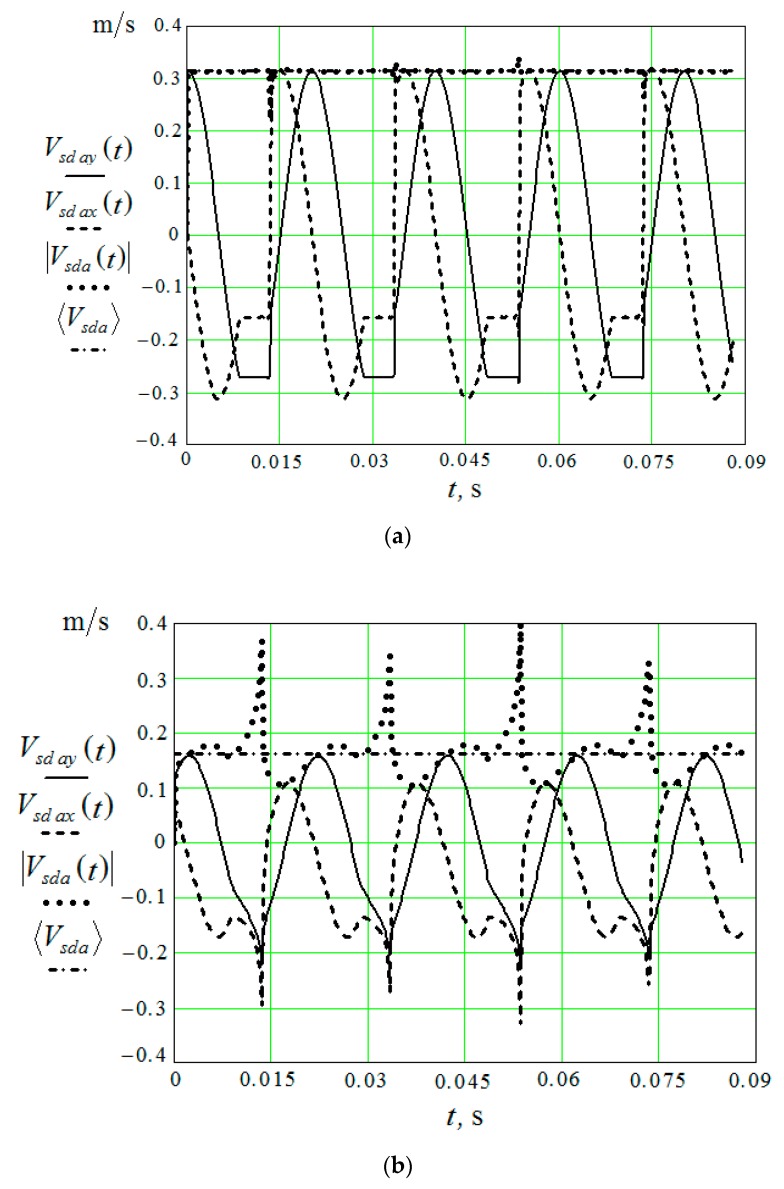
Horizontal and vertical component of the velocities of the pseudo-wave motion of the granules, the velocity modulus of the wave motion of the granules, the velocity of oscillatory movement of the granules under the action of an acoustic wave: (**a**) in the immediate vicinity of the reservoir working surface; and, (**b**) at a distance of 20 mm from the reservoir working surface.

To establish the value $\langle V_{sda}\rangle$, it is necessary to perform calculations while using Equations (14)–(30), which determine the vertical and horizontal components of the velocities of the pseudo-wave motion of the granules that were initiated by the working surface of the reservoir. The graphs of the wave motion velocity modulus—$\left|V_{sda}\left(t\right)\right|$ and $\langle V_{sda}\rangle$ calculated under the same conditions are also given here. The time-averaged velocity of the oscillatory motion of the granules under the action of an acoustic wave is also determined at a distance of 0 mm and 200 mm from the working surface of the vibrating machine reservoir (Figure 7). Calculations are made for the amplitude of the oscillations *A* = 1 mm and v=50 Hz (ω=314 radian/s) and are carried out with Mathcad.

It can be seen from the graphs that in the immediate vicinity from the reservoir working surface (Figure 7a) the values $\left|V_{sda}\left(t\right)\right|$ and $\langle V_{sda}\rangle$ practically coincide and they are independent of time, in spite of the fact that the vertical and horizontal components of the motion of the granules in the pseudo-wave are not of a harmonic character. These velocities are equal to the product of the amplitude of the oscillations of the reservoir and the circular speed of the engine of the vibration machine, which drives the reservoir working surfaces. At a considerable distance from the surface of the reservoir, the picture changes (Figure 7b), and averaging over time for the velocity modulus of the pseudo-wave motion becomes necessary.

**Figure 8:**
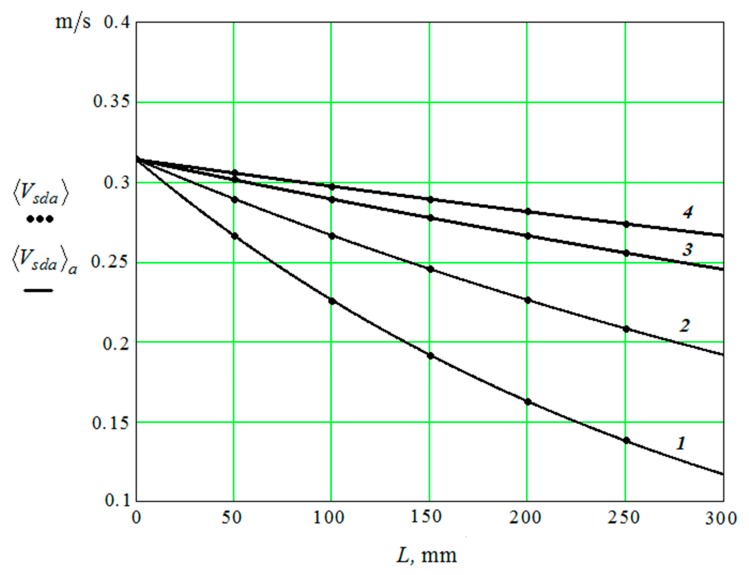
Dependence of the speed of granules movement $\langle V_{sda}\rangle$ on the distance to the reservoir working surface.

Points in Figure 8 show dependence $\langle V_{sda}\rangle$ on the distance to the working reservoir surface calculated by Equations (14)–(30).

The dependences of calculated values of $\langle V_{sda}\rangle$ on the distance to the plane of the reservoir and approximate curves ${\langle V_{sda}\rangle }_{a}$ of interpolation of values $\langle V_{sda}\rangle$ are shown by dots. The curves are presented for diameters of (d) granules: 1—10 mm; 2—20 mm; 3—40 mm; and, 4—60 mm.

The interpolation curve has the following form:

(45) $${\langle V_{sda}\rangle }_{a}=A_{s}\;\omega_{s}\;e^{-3.3\;y/10\;d}$$

The approximate curve is the result of interpolation of the calculated values (Figure 8). Dependences $\langle V_{sda}\rangle$ and ${\langle V_{sda}\rangle }_{a}$ practically coincide.

#### 3.2.3. The Movement of the Granules Near the Surface of the Oscillating Part

In the framework of the model used, we need to carry out the calculations analogous to those that were carried out in [2], while taking into account the real boundary conditions that are not described by harmonious functions, in order to determine the collective motion of granules under the action of the displacement of the part surface. However, the results obtained show that, immediately in the vicinity of the oscillating surface, the averaged velocity of the granules is equal to the product of the amplitude of the oscillations of the plane and the circular rotational speed of the engine of the vibration machine, which drives the reservoir working surfaces (see Figure 7a).

Thus, we can write the following relation:

(46) $$\langle V_{d}\rangle =A_{d}\;\omega_{d}$$

where $A_{d}$ and $\omega_{d}$ are the product of the amplitude of the oscillations of the reservoir surface and the rotational speed of the vibrating machine engine, respectively.

Proceeding from the Equations (42) and (43), the Equation (36) for the root-mean square velocity of chaotic motion—$\langle V_{x}\rangle$ near the part surface can be written in the following form:

(47) $$\begin{array}{ll} \langle V_{x}\rangle = & \sqrt{A_{s}{}^{2}\;\omega_{s}{}^{2}\;e^{-f_{1}\left(d\right)\;{\left(y-f_{2}\left(d\right)\right)}^{2.99}}+\frac{3}{4}\;\gamma {\left(A_{s}\;\omega_{s}\;e^{-3.3y/10d}+A_{d}\;\omega_{d}\right)}^{2}}+\\ & +\frac{\sqrt{3\gamma }}{2}\;\left(A_{s}\;\omega_{s}\;e^{-3.3y/10d}+A_{d}\;\omega_{d}\right) \end{array}$$

## 4. The Removal of Metal from the Part Surface as A Result of Multi-Agent Group Action of the Oscillating Reservoir Surface and the Part

The expression for determining the removal of metal from the surface of a part, depending on the frequency and amplitude of oscillations of the part surface and the reservoir, is known [21]. However, for our case, this expression is unacceptable, since it assumes a processing mode in the conditions of immovable fixation of the part.

In the case under consideration, when the autonomous movement of both the reservoir working surface and the surface of the processed parts simultaneously takes place, it is necessary to determine the rate of chaotic movement of the granules, which is proportional to the product of the amplitude and angular velocity of oscillations of the plane wall of the reservoir.

It is obvious that, in our case, the speed of chaotic motion of molecules near the part surface is equal $\langle V_{x}\rangle$; therefore, the formula for metal removal will be as follows:

(48) $$\begin{array}{ll} Q= & \frac{C_{\Sigma }\;R\;{\langle P_{m}\rangle }^{0.875}\;\left(1-k_{\omega d}\omega_{d}{}^{2}-k_{\omega s}\omega_{s}{}^{2}+k_{Ad}A_{d}{}^{2}+k_{As}A_{s}{}^{2}\right)}{\left(1+C\;{\langle V_{x}\rangle }^{2}\right)}\times \\ & \times {\langle V_{x}\rangle }^{2}\;F\left(k\right)s_{pro}t \end{array}$$

Here, F(k) and $s_{pro}$ is a function that depends on the coefficient of friction and the surface area of the processed parts; $C_{\Sigma }$ and $\langle P_{m}\rangle$ is a combined constant, which is determined by the mechanism of “cutting” by a granule of metal from the surface of the processed part and by the pressure exerted by the group of pseudo-gas granules on the processed part surface; $k_{\omega s},\overset{}{}k_{\omega d},\overset{}{}k_{As},\overset{}{}k_{Ad}$ are the coefficients of the speed $\langle V_{x}\rangle$ corrections to the circulation motion of the granules according to the parameters of the frequency and amplitude of the vibrations of the reservoir working surface and the part; $C\approx \left(10-30\right)\frac{c^{2}}{m^{2}}$; R is the average radius of the abrasive granule; and, t is the time of vibration processing.

The single medium granule value, averaged over the pressure $\langle P_{m}\rangle$ of cross-section of the reservoir, is determined by the relation:

(49) $$\langle P_{m}\rangle =\frac{C_{p}rA\omega }{\left(1+CA^{2}\omega^{2}\right)}{\left(1-\varepsilon \right)}^{{}^{\frac{3\sqrt{2}Lr}{2R\left(1+CA^{2}\omega^{2}\right)}}}$$

where r is the filling factor of the volume with granules (≈0.66) and R is the radius of the abrasive granules.

Function *F*(*k*) is determined by the following expression:

(50) $$\begin{array}{ll} F\left(k\right)= & \frac{\left(\cos\left({\mathrm{tn}}^{-1}\left(7k\right)\right)-\frac{7}{2}k\left(1-\sin\left({\mathrm{tn}}^{-1}\left(7k\right)\right)\right)\left(1-\sin\left({\mathrm{tn}}^{-1}\left(7k\right)\right)\right)\right)}{\left(\frac{\pi }{2}-{\mathrm{tn}}^{-1}\left(7k\right)\right)}+\\ & +\frac{{\left(1-\cos\left({\mathrm{tn}}^{-1}\left(7k\right)\right)\right)}^{2}}{7k\left({\mathrm{tn}}^{-1}\left(7k\right)\right)} \end{array}$$

Function F(k) is derived from the assumption that the reflection of the granule from the processed part surface in the direction perpendicular to its surface is absolutely elastic, that is, without the loss of kinetic energy. Taking into account the recovery coefficient and Equations (9)–(13), we assume that k=0.5(1+β)f [18].

**Figure 9:**
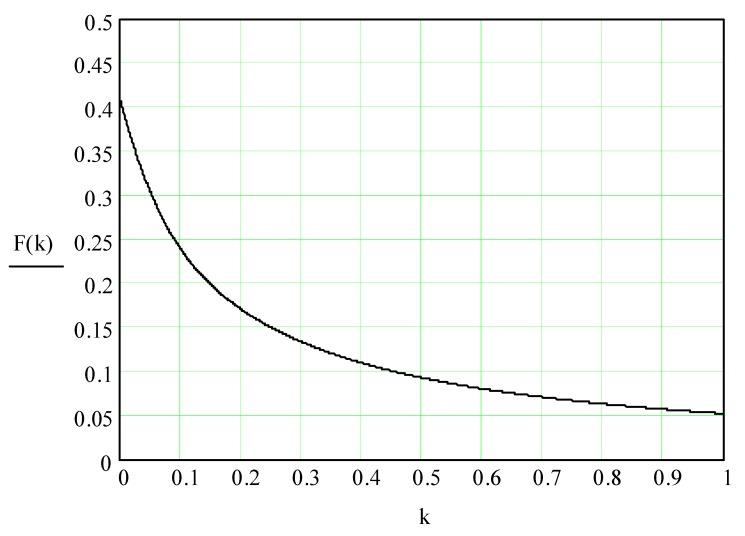
Behavior of function F(k) as a function of the parameter k=0.5(1+β)f.

The Equation (50) is illustrated by a curve (Figure 9).

Figure 10, Figure 11, Figure 12, Figure 13, Figure 14, Figure 15, Figure 16, Figure 17, Figure 18 and Figure 19 show the results of calculations of the metal removal, which were obtained from Equation (48).

Analysis of the metal removal during the processing of parts with multi-energy vibration processing technology allowed for us to make the following comments to the obtained graphical dependencies when varying the parameters of amplitude and frequencies of oscillations of the reservoir surface and the processed part, as well as the distance between them and the size of the granules of the working medium used.

It is established that the oscillatory movements of the part surface and the vibrating machine reservoir provide the energy of the movement of the granules necessary for the machining of the part. In this case, if the part surface transmits the force impulse directly to the adjacent granules, the transfer of kinetic energy from the reservoir surface to the granules of the abrasive located in the immediate vicinity of the part surface should occur through two “channels”:

—the first “channel” transmits the impulse of chaotic movement of the granules—$\langle V_{sd}\rangle$. In this case, the dependence of its magnitude on the distance to the reservoir surface is shown graphically (Figure 4); and,

—the second “channel” for impulse transmission determines the pseudo-wave motion of the working medium granules. The dependence of the time-averaged velocity of the oscillatory motion of the granules under the action of a pseudo-acoustic wave—$\langle V_{sda}\rangle$ is mapped (Figure 8).

**Figure 10:**
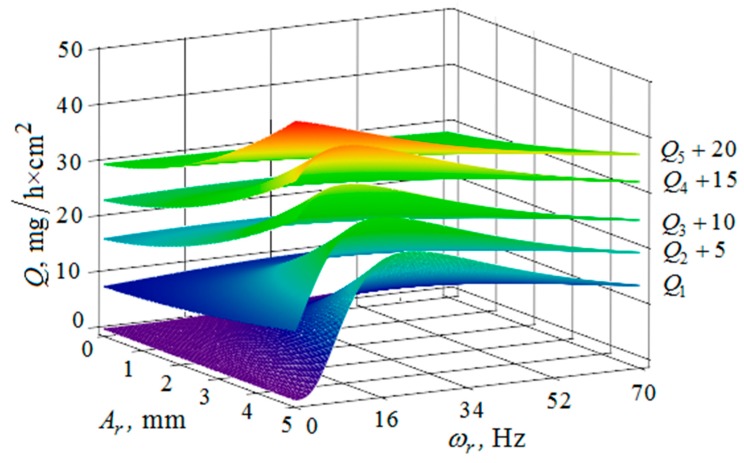
Dependence of the metal removal Q, mg/h·cm^2^ on the amplitude of $A_{r}$, mm and the oscillation frequency $\omega_{r}$, Hz of the reservoir working surface for different values of the vibration frequency of the processed part surface: ($Q_{1}$, $Q_{2}$, $Q_{3}$, $Q_{4}$, $Q_{5}$—metal removal at $\omega_{d}$ = 0; 16; 34; 52; 70 Hz); amplitude $A_{d}$ of the oscillation of the part surface 1 mm.

**Figure 11:**
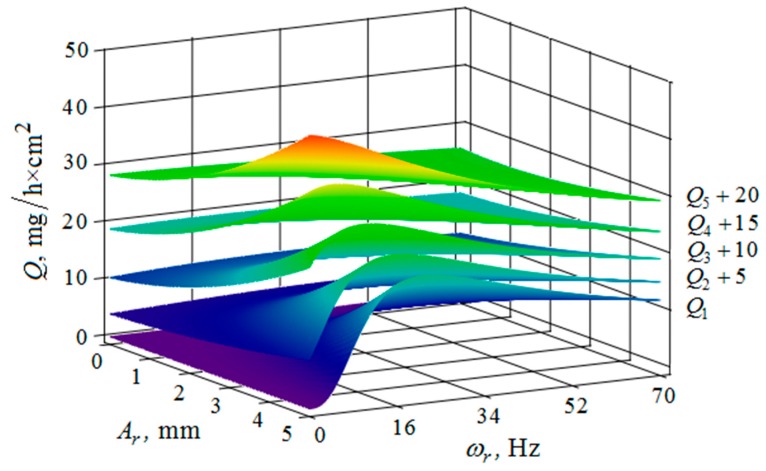
Dependence of the metal removal Q, mg/h·cm^2^ on the amplitude of $A_{r}$,mm and the oscillation frequency $\omega_{r}$, Hz of the reservoir working surface for different values of the amplitude of oscillations of the part surface: ($Q_{1}$, $Q_{2}$, $Q_{3}$, $Q_{4}$, $Q_{5}$—metal removal at $A_{d}$ = 1; 2; 3; 4; 5 mm); frequency of oscillations $\omega_{r}$ of the reservoir surface 16 Hz.

**Figure 12:**
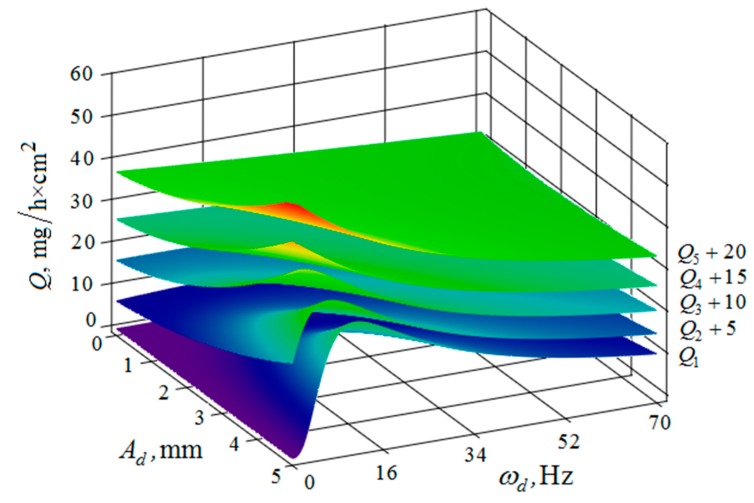
Dependence of the metal removal Q, mg/h·cm^2^ on the amplitude of $A_{d}$, mm and frequency $\omega_{d}$, Hz of oscillations of the processed part surface for various values of the amplitude of oscillations of the reservoir working surfaces: ($Q_{1}$, $Q_{2}$, $Q_{3}$, $Q_{4}$, $Q_{5}$—metal removal at $A_{r}$ = 1; 2; 3; 4; 5 mm); frequency $\omega_{r}$ of oscillations of the reservoir working surface 50 Hz.

**Figure 13:**
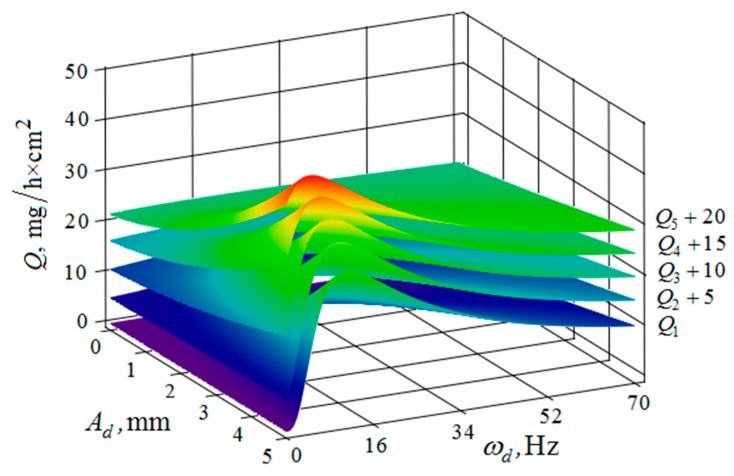
Dependence of the metal removal Q, mg/h·cm^2^ on the amplitude $A_{d}$, mm and frequency $\omega_{d}$, Hz of oscillations of the processed part surface for various values of the vibration frequency of oscillations of the reservoir working surfaces: ($Q_{1}$, $Q_{2}$, $Q_{3}$, $Q_{4}$, $Q_{5}$—metal removal at $\omega_{r}$ = 0; 16; 34; 52; 70 Hz); the amplitude $A_{r}$ of oscillation of the working reservoir surface is 1 mm.

**Figure 14:**
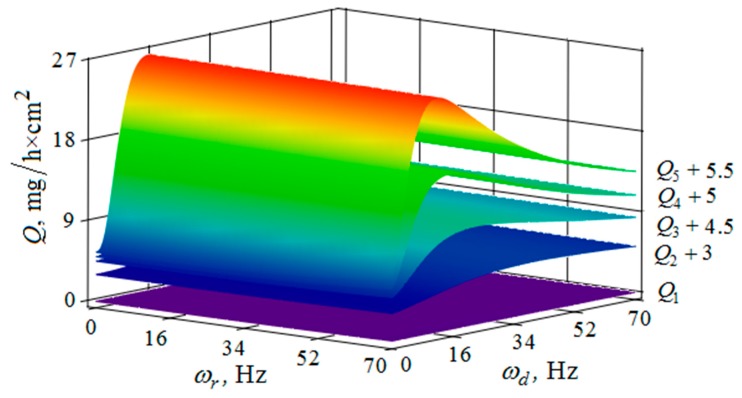
Dependence of the metal removal Q, mg/h·cm^2^ on frequencies of oscillations $\omega_{r}$, Hz and $\omega_{d}$, Hz of the reservoir working surface and the processed part for different values of the amplitude of the oscillations of the part: ($Q_{1}$, $Q_{2}$, $Q_{3}$, $Q_{4}$, $Q_{5}$—metal removal at $A_{r}$ = 1; 2; 3; 4; 5 mm); the amplitude $A_{r}$ of oscillation of the reservoir working surface is 1 mm.

**Figure 15:**
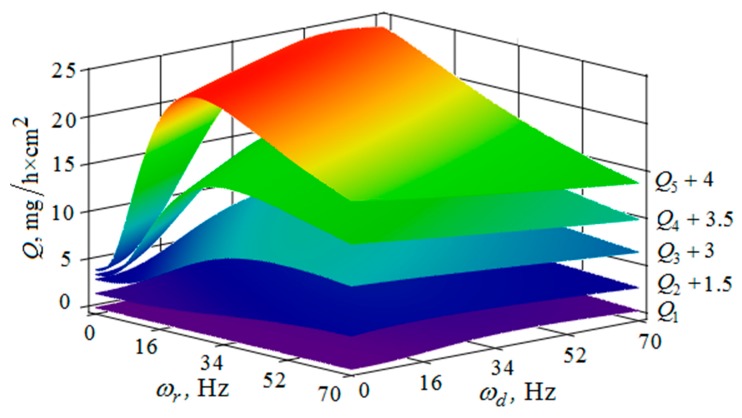
Dependence of the metal removal Q, mg/h·cm^2^ on frequencies oscillations $\omega_{r}$, Hz and $\omega_{d}$, Hz of the reservoir working surface and the processed part for different values of the amplitude of the oscillations of the reservoir working surface: ($Q_{1}$, $Q_{2}$, $Q_{3}$, $Q_{4}$, $Q_{5}$—metal removal at $A_{d}$ = 1; 2; 3; 4; 5 mm); the amplitude $A_{d}$ of oscillations of the part surface 1 mm.

**Figure 16:**
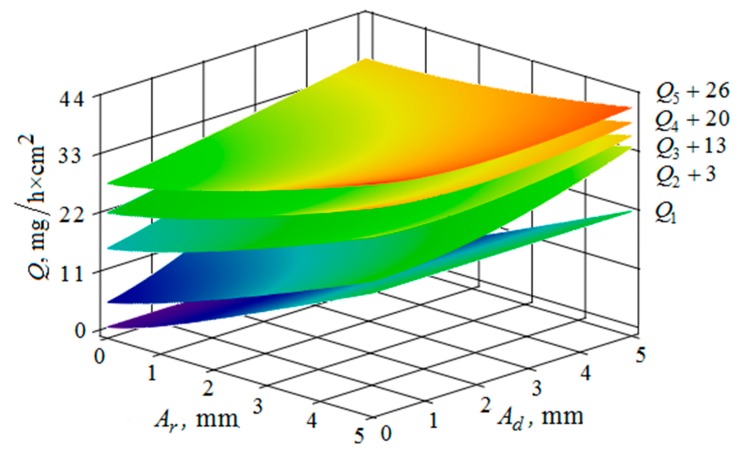
Dependence of the metal removal Q, mg/h·cm^2^ on the amplitudes of oscillations $A_{r}$, mm and $A_{d}$, mm of the reservoir working surface and the processed part for different values of frequencies of oscillations of the part surface: ($Q_{1}$, $Q_{2}$, $Q_{3}$, $Q_{4}$, $Q_{5}$—metal removal at $\omega_{d}$ = 0; 16; 34; 52; 70 Hz); the oscillation frequency $\omega_{r}$ of the reservoir working surface is 50 Hz.

**Figure 17:**
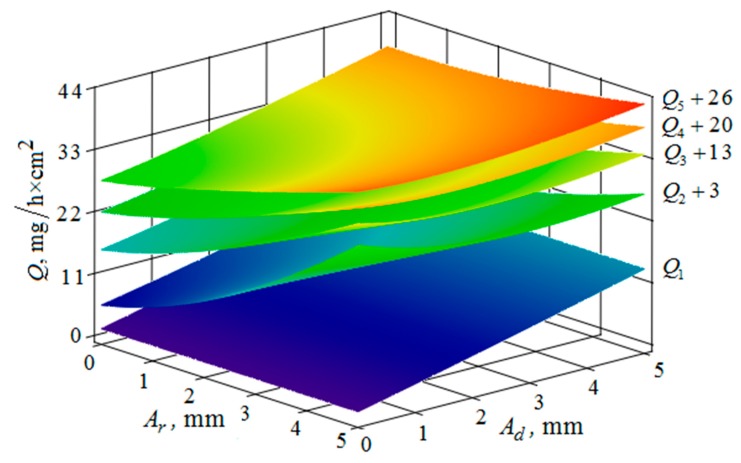
Dependence of the metal removal Q, mg/h·cm^2^ on the amplitudes of oscillations $A_{r}$, mm and $A_{d}$, mm of the reservoir working surface and the processed part for different values of frequencies of oscillations of the working reservoir surface: ($Q_{1}$, $Q_{2}$, $Q_{3}$, $Q_{4}$, $Q_{5}$—metal removal at $\omega_{r}$ = 0; 16; 34; 52; 70 Hz); the frequency $\omega_{d}$ of oscillations of the part surface 50 Hz.

**Figure 18:**
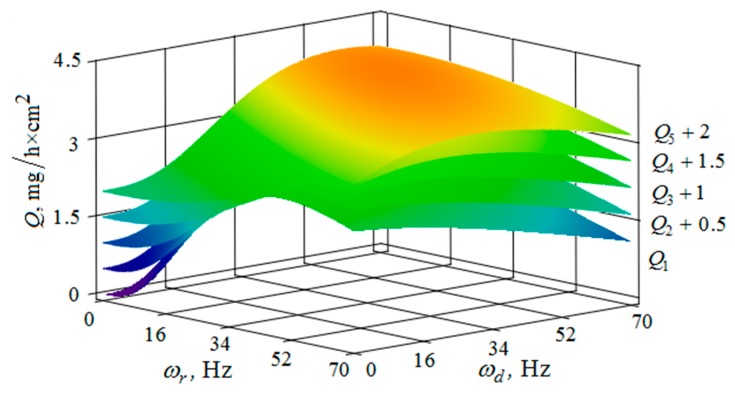
Dependence of the metal removal Q, mg/h·cm^2^ on the frequencies of oscillations $\omega_{r}$, Hz and $\omega_{d}$, Hz of the reservoir working surfaces and the processed part for different values of the distance between them: ($Q_{1}$, $Q_{2}$, $Q_{3}$, $Q_{4}$, $Q_{5}$—metal removal at Y = 40; 80; 120; 160; 200 mm); the amplitudes of the oscillations of the reservoir surface $A_{r}$ and the processed part $A_{d}$ planes are 1 mm.

**Figure 19:**
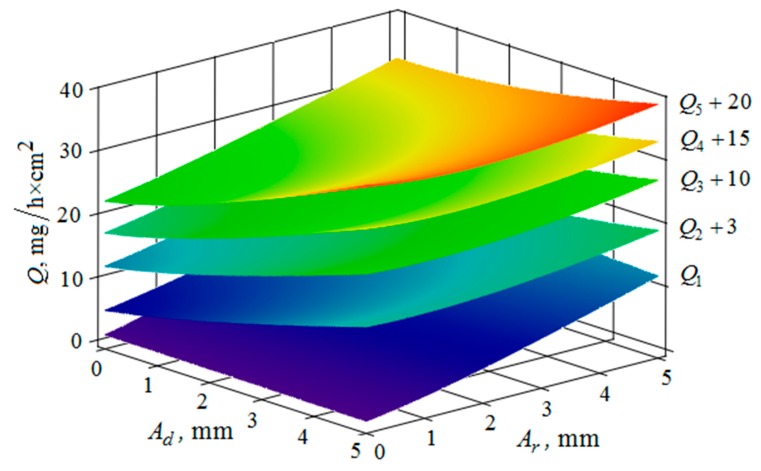
Dependence of the metal removal Q, mg/h·cm^2^ on the amplitudes of oscillations $A_{r}$, mm and $A_{d}$, mm of the reservoir working surfaces and the processed part for different values of the distance between them: ($Q_{1}$, $Q_{2}$, $Q_{3}$, $Q_{4}$, $Q_{5}$—metal removal at Y = 40; 80; 120; 160; 200 mm); the frequencies of oscillations of the reservoir $\omega_{r}$ working surfaces and the part $\omega_{d}$ surface 50 Hz.

**Figure 20:**
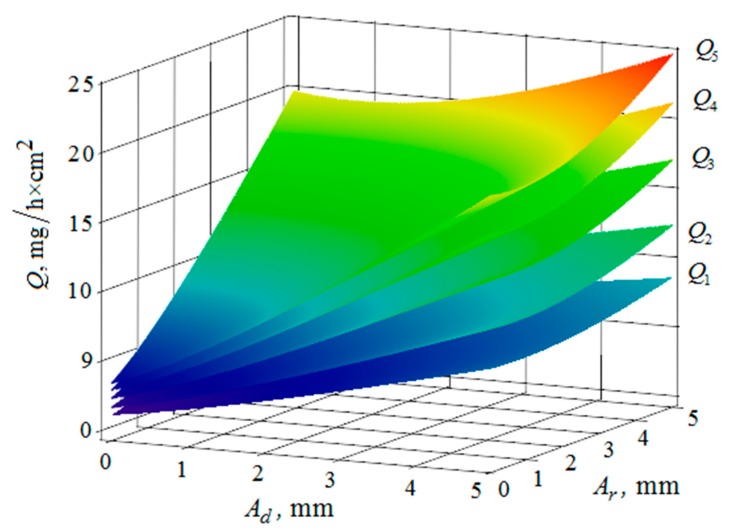
Dependence of the metal removal Q, mg/h·cm^2^ on the amplitudes of oscillations $A_{r}$, mm and $A_{d}$, mm of the reservoir working surfaces and the processed part for different values of diameters of the abrasive granules: ($Q_{1}$, $Q_{2}$, $Q_{3}$, $Q_{4}$, $Q_{5}$—metal removal at d = 10; 15; 20; 25; 30 mm); the frequencies of oscillations of the reservoir $\omega_{r}$ working surfaces and the part $\omega_{d}$ surface 50 Hz.

**Figure 21:**
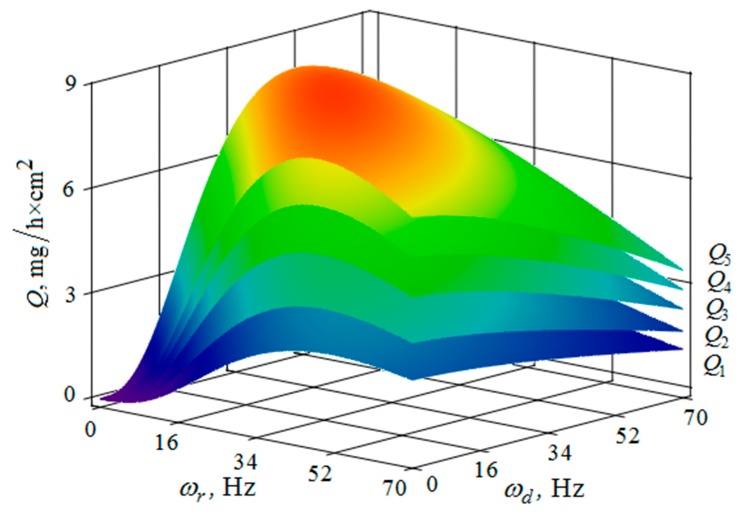
Dependence of the metal removal Q, mg/h·cm^2^ on the frequencies of oscillations $\omega_{r}$, Hz and $\omega_{d}$, Hz of the reservoir working surfaces and the processed part surface for different values of the diameters of the abrasive granules: ($Q_{1}$, $Q_{2}$, $Q_{3}$, $Q_{4}$, $Q_{5}$—metal removal at d = 10; 15; 20; 25; 30 mm); amplitudes of oscillations of the reservoir $A_{r}$ working surfaces and the part $A_{d}$ 1 mm.

The quantitative relationships that were obtained in the work make it possible to calculate the efficiency of metal removal in the case of multi-energy vibration processing of parts, depending on the amplitudes and vibration frequencies of the part and vibrating machine, the distance from the reservoir wall to the processed parts surface, and the diameter of the abrasive medium granules. The total multi-agent action on the vibration processing process is graphically shown (Figure 10, Figure 11, Figure 12, Figure 13, Figure 14, Figure 15, Figure 16, Figure 17, Figure 18, Figure 19, Figure 20 and Figure 21). On all graphical dependencies, various constants are added to the values of metal removal to improve visual perception. The constants are shown for each surface. Without such an operation, the dependencies can partially merge with each other.

The metal removal Q, depending on the amplitude $A_{r}$ and the oscillation $\omega_{r}$ frequency of the reservoir surface for various values of the amplitude $A_{d}$ and frequency $\omega_{d}$ of oscillations of the part surface is graphically shown (Figure 10 and Figure 11). It can be seen from the dependences that for small values of the amplitude and frequency of the oscillations of the part surface, the dependence of the metal removal on the oscillation frequency of the reservoir surface reaches its maximum. The amount of metal removal decreases with an increase in the amplitude and frequency of oscillations of the part surface and the frequency of oscillations of the reservoir surface. This is connected with the redistribution of the vibrational energy into the energy of the granules’ motion, that is, when the energy of the chaotic movement of the working medium granules decreases, the metal removal is also decreased.

The metal removal Q depending on the amplitude $A_{d}$ and frequency $\omega_{d}$ of the oscillation of the part surface for different values of the amplitude and frequency of oscillations of the reservoir surface is graphically shown (Figure 12 and Figure 13). The nature of the dependence of metal removal (Figure 12) is similar to the previously presented trends (Figure 10 and Figure 11). The dependences have some differences, which result from the fact that the metal removal maximum is manifested at all values of the oscillation frequencies of the reservoir surface (Figure 13). This is due to the weakening of the propagation of oscillations as they move away from the reservoir surface to the part surface.

The metal removal Q depending on the oscillation $\omega_{r}$ frequencies and of the reservoir surface the $\omega_{d}$ part surface for different values of the oscillation amplitude of the part surface and the reservoir surface is graphically shown (Figure 14 and Figure 15). The dependence of the metal removal on the oscillation $\omega_{r}$ frequency is practically absent (Figure 14). This is due to the damping of the oscillations of the reservoir surface as they propagate from the reservoir surface to the part. The dependence of the metal removal on the frequency of oscillations of the part surface has an ordinary character with a clearly expressed maximum. As the amplitude of the oscillations of the reservoir surface increases, the influence of the frequency of their oscillations on the metal removal begins to appear (Figure 15). The non-monotonic dependence of the metal removal on the vibration frequency of the $\omega_{d}$ part surface is not manifested due to the small value of the oscillation amplitudes of the part.

Thus, proceeding from the regularities graphically shown (Figure 10, Figure 11, Figure 12, Figure 13, Figure 14 and Figure 15), it can be concluded that in the case of multi-energy treatment the combined action of the frequencies and amplitudes of vibrations of the vibration machine reservoir surfaces and the processed part surface exerts influence upon the distribution of kinetic energy between the movement of granules and their chaotic movement.

The metal removal Q, depending on the amplitudes $A_{r}$ of oscillations and of the reservoir wall and $A_{d}$ part surface is graphically shown (Figure 16 and Figure 17) for different values of vibration frequencies of the surfaces of the part and the vibrating machine reservoir. The dependences of the metal removal on the amplitudes are of monotonic character. At the same time, the dependence of metal removal on the oscillation frequencies of the part and the reservoir surface is not monotonic. This is manifested in the need to sharply increase the values of the constants added to the values of metal removal to facilitate visual perception of the dependencies, which begins at a frequency of 34 Hz.

The metal removal Q, depending on the frequencies and amplitudes of oscillations of the reservoir surface and the part surface, is graphically shown for different values of the Y distances between the surfaces of the reservoir and the part (Figure 18 and Figure 19). These dependences qualitatively coincide with those given earlier (Figure 15 and Figure 16). On the graph, the presence of the optimum relationships between the oscillation frequencies of the reservoir surface and the part is clearly visible (Figure 18). It ensures maximum metal removal. The dependences illustrate the monotonous increase in the metal removal rate as the amplitude of oscillations of the reservoir surface and the part surface increases (Figure 19).

The metal removal Q dependence on the amplitude and frequency of vibrations of the reservoir surface and the part is graphically shown (Figure 20 and Figure 21) for different values of diameters of abrasive granules. The results show that, as in the previous graphs, the maximum value of metal removal is achieved at some optimum values of vibration frequencies of the reservoir surface and the part (Figure 21). The dependence illustrates the situation when the maximum value of metal removal is realized at the maximum values of the oscillation amplitudes of the reservoir surface and the part (Figure 20).

It should be separately noted that, when constructing metal removal surfaces (Figure 20 and Figure 21), we did not have to use the method of adding some constants to improve the visual perception of the graphs, as was done earlier (Figure 10, Figure 11, Figure 12, Figure 13, Figure 14, Figure 15, Figure 16, Figure 17, Figure 18 and Figure 19). This is a consequence of a sharp decrease in the frictional losses of kinetic energy at the moment of mutual collisions of the granules of the working medium during the transfer of the force impulse from the reservoir surface to the part surface as the granule sizes increase (Figure 4 and Figure 9). This explains the use of a mixture of the working medium abrasive granules with two different sizes but the same masses, if vibration processing of the part surface is required with the help of small size granules. The granules of large size abrasive in such a medium work as “distributors” of a power pulse with smaller frictional losses than the granules of small dimensions during mutual collisions. The identity of the masses of large and small granules is explained by the fact that the transfer of momentum from large abrasive granules to small granules will be the highest possible in this case.

## 5. Conclusions

The modelling of processes occurring during multi-energy vibration processing of parts, conducted on the basis of the application of the kinetic theory of gases, makes it possible to find the optimal combination of various process parameters in terms of maximizing the intensity of metal removal. Among these parameters are: frequencies and amplitudes of oscillations of the vibration machine reservoir surface and the surface of the processed part; the distance between them; and, the size of the granules.

It is quite clear that the whole variety of shapes of processed parts cannot be quantitatively described by this model, because of the simplicity that was used as a basis for the design scheme (the reservoir surface and the parallel flat part surface). At the same time, the search for optimal relationships between the parameters that characterize the process of vibration processing, for each particular shape of the processed parts, requires additional experimental studies. However, the developed model establishes the mutual influence of the main parameters of vibration processing on its efficiency. Thus, the model makes it possible to carry out experiments to optimize the process while taking into account the influence of all the characteristics and, consequently, its application will significantly reduce the time and material costs of conducting such experiments.

An analysis of experimental studies of the vibration multi-energy processing, which consist in joint action of the forces on the working medium and the processed part, made it possible to establish a numerical increase in the mass metal removal by 1.6–1.8 times in comparison with traditional vibration processing, in which there is no independent action on the working medium of autonomously oscillating processed parts. In addition, the use of multi-energy processes makes it possible to establish the dependence of the weight metal removal on the vibration modes of the working surfaces reservoir and the processed parts, as well as their distances from these surfaces and the size of the abrasive working medium granules being used. The problems that are considered in this article can be used in the development of draft standards governing the functional indicators of finishing and grinding vibrating machines.
